# The Predicted Influence of Climate Change on Lesser Prairie-Chicken Reproductive Parameters

**DOI:** 10.1371/journal.pone.0068225

**Published:** 2013-07-11

**Authors:** Blake A. Grisham, Clint W. Boal, David A. Haukos, Dawn M. Davis, Kathy K. Boydston, Charles Dixon, Willard R. Heck

**Affiliations:** 1 Department of Natural Resources Management, Texas Tech University, Lubbock, Texas, United States of America; 2 U.S. Geological Survey, Texas Cooperative Fish and Wildlife Research Unit, Texas Tech University, Lubbock, Texas, United States of America; 3 U.S. Geological Survey, Kansas Cooperative Fish and Wildlife Research Unit, Kansas State University, Manhattan, Kansas, United States of America; 4 Department of Fish and Wildlife Resources, University of Idaho, Moscow, Idaho, United States of America; 5 Texas Parks and Wildlife Department, Austin, Texas, United States of America; 6 Wildlife Plus Consulting, Alto, New Mexico, United States of America; 7 Grasslans Charitable Foundation, Causey, New Mexico, United States of America; University of Sydney, Australia

## Abstract

The Southern High Plains is anticipated to experience significant changes in temperature and precipitation due to climate change. These changes may influence the lesser prairie-chicken (*Tympanuchus pallidicinctus*) in positive or negative ways. We assessed the potential changes in clutch size, incubation start date, and nest survival for lesser prairie-chickens for the years 2050 and 2080 based on modeled predictions of climate change and reproductive data for lesser prairie-chickens from 2001–2011 on the Southern High Plains of Texas and New Mexico. We developed 9 *a priori* models to assess the relationship between reproductive parameters and biologically relevant weather conditions. We selected weather variable(s) with the most model support and then obtained future predicted values from climatewizard.org. We conducted 1,000 simulations using each reproductive parameter’s linear equation obtained from regression calculations, and the future predicted value for each weather variable to predict future reproductive parameter values for lesser prairie-chickens. There was a high degree of model uncertainty for each reproductive value. Winter temperature had the greatest effect size for all three parameters, suggesting a negative relationship between above-average winter temperature and reproductive output. The above-average winter temperatures are correlated to La Niña events, which negatively affect lesser prairie-chickens through resulting drought conditions. By 2050 and 2080, nest survival was predicted to be below levels considered viable for population persistence; however, our assessment did not consider annual survival of adults, chick survival, or the positive benefit of habitat management and conservation, which may ultimately offset the potentially negative effect of drought on nest survival.

## Introduction

The lesser prairie-chicken has experienced as much as a 97% decline in population size [1] and similar suspected declines in occupied area from historic levels. The species is currently proposed as threatened under the United States Endangered Species Act and is a priority species under the Great Plains Landscape Conservation Cooperative (GPLCC). The semi-arid region of the Southern High Plains encompasses the entire distribution of lesser prairie-chickens, which is considered a principal indicator species of the ecosystem. Previous studies have examined the reproductive ecology of lesser prairie-chickens on the Southern High Plains [2], [3], [4], [5] and similar studies are currently being conducted elsewhere across the species distribution. However, the influence of drought and climate change on lesser prairie-chicken reproductive ecology has, to date, been largely overlooked. This is of concern, as lesser prairie-chickens appear to be particularly sensitive to landscape alterations [6], [7] and drought [8], [9]. Drought is suspected to negatively influence prairie grouse through reduced growth of vegetation that provides nesting, roosting, and escape cover, and food [10], [11]. Furthermore, there is evidence that home range sizes increase [10], [12] and recruitment is lower during drought years [10], [13]. Home range size expansion during drought years may lead to localized abandonment, especially in fragmented landscapes. Furthermore, landscape alterations and management (e.g., herbicide treatment of shrubs, grazing systems) appear to influence resource selection, survival, and reproductive success of lesser prairie-chicken populations.

One of the primary science goals for the Landscape Conservation Cooperatives and the U. S. Geological Survey National Climate Change and Wildlife Science Center is to assess the vulnerability and risk of species to climate change. The issue of climate change as a challenge to bird conservation in arid and semi-arid regions also was identified by federal and state fish and wildlife management agencies as a high priority issue. A key issue in conservation of lesser prairie-chickens in context of climate change is the lack of estimates for many specific vital rates and sufficient sample sizes. Despite substantial efforts to conserve lesser prairie-chickens and their habitat, long-term studies that provide adequate data to properly allow for predictive modeling of the role climate change may have on the reproductive ecology of this species are lacking. This is important in that the Great Plains is anticipated to experience increased maximum and minimum temperatures, increased intensity but reduced occurrence of precipitation events, and spring and the associated environmental phenology occurring earlier [14]. In particular, climate change forecasts indicate the Southern High Plains will become drier with more frequent extreme heat events and decreased precipitation events [15].

Increased surface and ambient temperature may lead to egg death and/or nest abandonment. This may be exacerbated if, due to low precipitation, nesting phenology shifts to later in the year when temperatures are increased. Thus, lesser prairie-chickens will be exposed to increased temperatures that may exceed both thresholds of egg survival and tolerances of adults. These climate change predictions may have long-term negative impacts on lesser prairie-chicken populations in these regions as survival of chicks from hatch to the next breeding season has been identified as the key demographic parameter affecting lesser prairie-chicken populations [16].

Our goals were two-fold. First, was an analysis and assessment of an 11-year data set of lesser prairie-chicken reproductive ecology in response to seasonal weather conditions. This included documentation of clutch size, incubation start date, and nest survival. Second, we assessed these parameters in context of projected climate change scenarios for the region. We believe our study will provide a compilation and analysis of existing phenological data from a long-term data set and an assessment of reproductive data that can be used to develop predictive models of response to future climate change.

## Materials and Methods

### Ethics Statement

All methods were approved under Texas Tech University Institutional Animal Care and Use Protocol #1052-08. Any use of trade names or products does not constitute endorsement by the U.S. Government. We thank a multitude of private land owners in Texas for private land access. We thank Jim Weaver and all members of the Grasslans Charitable foundation for providing financial and logistical support and study site access in New Mexico. We thank Apple Wood and a plethora of field technicians for field data collection in New Mexico and Texas. We thank the Sutton Avian Research Center for data collection on the New Mexico study area from 2001–2005. Financial and logistical support was provided by Texas Tech Department of Natural Resources Management, United States Geological Survey, Texas Parks and Wildlife, New Mexico Game and Fish, Great Plains Landscape Conservation Cooperative, and The Nature Conservancy.

### Capture

We captured subadult and adult lesser prairie-chickens on leks during spring (March–April) using walk-in funnel traps [17], rocket nets, and drop-nets from 2001–2011. We recorded gender, age, lek of capture, time of capture, weight, wing cord (mm), pinnae length (mm), and tail length (mm) for each bird. We determined gender using pinnea length, presence of an eye comb, tail feather markings, and other plumage characteristics [12]. We determined age by plumage characteristics; subadults have white spotting within 2.5 cm of the tip of the 9^th^ and 10^th^ primaries, whereas white spotting is absent within 2.5 cm of primary tips in adult birds [12]. We banded each female with either a New Mexico Department of Game and Fish or a Texas Parks and Wildlife Department aluminum blunt-end leg band. We equipped each bird with a 13 gram loop style radio-transmitter (American Wildlife Enterprises, Florida, USA; Advanced Telemetry Systems, Minnesota, USA) with an eight-hour mortality sensor and then released them at the capture site.

### Nest Location

We determined nest locations by approaching the hen via homing when locations remained unchanged daily between two subsequent location attempts. We revisited a nest only if the hen was determined via telemetry to be off the nest; nest revisits were to assess the status of the nest (e.g., depredated, hatched, abandoned). At each nest, we counted the number of eggs present at the initial flush and each subsequent visit (when applicable). We calculated nest initiation by backdating from the start of incubation 1 day for each egg in nest [18]. We made all attempts to reduce observer disturbance to the nest site.

### Weather Data Collection and Modeling

A digital recording rain gauge (Rainwise, Incorporated; Harbor, Maine, United States) was established on each study area to monitor precipitation. In addition, a weather station was placed on each study area to monitor daily weather conditions. We calculated the mean temperature and total rainfall for each month within each year of the study. We compiled the data and modeled clutch size, incubation initiation, and nest survival as a function of seasonal weather conditions. Because our interest was the influence of seasonal conditions, we did not model reproductive parameters as a function of daily weather variables; rather we averaged temperatures and precipitation across biological meaningful seasons (Table S1). We did not include any renest attempts from the incubation analysis because timing of renests is dependent on failure of the initial nest, and may influence these attempts more than seasonal weather patterns.

We developed seasonal weather models that were most likely to affect percent vegetation cover, food availability, and physiology. For example, we incorporated winter and spring precipitation and temperature because they were most likely to reflect food availability and short-term cover for nesting; shinnery oak (*Quercus harvardii*) and sand sagebrush (*Artemisia filifolia*) leaf out is determined by winter and spring conditions [4]. We incorporated yearly and wet-season precipitation as a parameter affecting cover, because sand shinnery oak has the ability to store large amounts of water over long periods of time [19] and lesser prairie-chickens nest in residual grass cover from previous growing seasons. Spring and winter temperature and precipitation may affect reproductive ecology disproportionately compared to other parameters [20]. Therefore, we included these as individual parameters to assess per unit change in reproductive parameter for each environmental variable given in each linear equation for each factor acting individually as well as acting as additive factors.

We used Akaike’s Information Criterion (AIC) [21] to assess the role of seasonal weather conditions on clutch size and incubation initiation. We modeled clutch size and incubation initiation, as a function of yearly precipitation, wet season precipitation, winter temperature, winter precipitation, spring temperature, spring precipitation, winter severity (temperature and precipitation), spring severity (temperature and precipitation), and a global model that incorporated all parameters (Table S2).

We modeled daily nest survival [22] as a function of the same nine seasonal *a priori* models used to model clutch size and incubation initiation. We used the logit-link function in program MARK [23] to model daily survival rates. Weather data were not collected in 2000, one year before the New Mexico project was initiated. Therefore, yearly rainfall, wet season rainfall, winter temperatures and winter precipitation were not available for the 2001 nesting season. We searched for supplementary weather data (www.mesonet.ttu.edu) but these data were not available for the study area. Therefore, in 2001, we used the mean of the variable of interest as a surrogate for missing data in program MARK.

### Statistical Analysis

For each model, we obtained the residual sums of squares and then calculated Akaike’s Information Criterion for small samples (AIC*c*), changes in AIC*c* and ΔAIC*c* values, and Akaike weights (AIC*w*). We used the preceding model selection criteria to evaluate model performance and select the best approximating model [24]. For each analysis, we selected the individual parameters that had the most support from Akaike’s Information Criterion. If there was no overwhelming support for any parameter, we modeled the change in reproductive parameter as a function of all seasonal weather parameters in the global model.

### Future Forecasting

We obtained future climatic forecasts from www.climatewizard.org. Climatewizard.org is a web-based, spatial tool that allows users to obtain downscaled future forecast of changes of average temperature and precipitation for the years 2050 and 2080. Climate wizard allows users to obtain future forecasts from three carbon dioxide (CO_2_) emission models (B1, A1B, A2; Table S3; [25]) and 16 Atmospheric and Oceanic General Circulation Models (AOGCMs). Users can select three scenarios within each CO_2_ emission model: best case, worst case, and ensemble averages. The worst case scenario estimates the largest change projected by each general circulation model across each CO_2_ model. The best case scenario estimates the smallest change projected by each general circulation model across each CO_2_ model. The ensemble model estimates the value where half of the models projected the largest change and the other half projects the smallest change for each general circulation model across each CO_2_ model.

**Figure 1:**
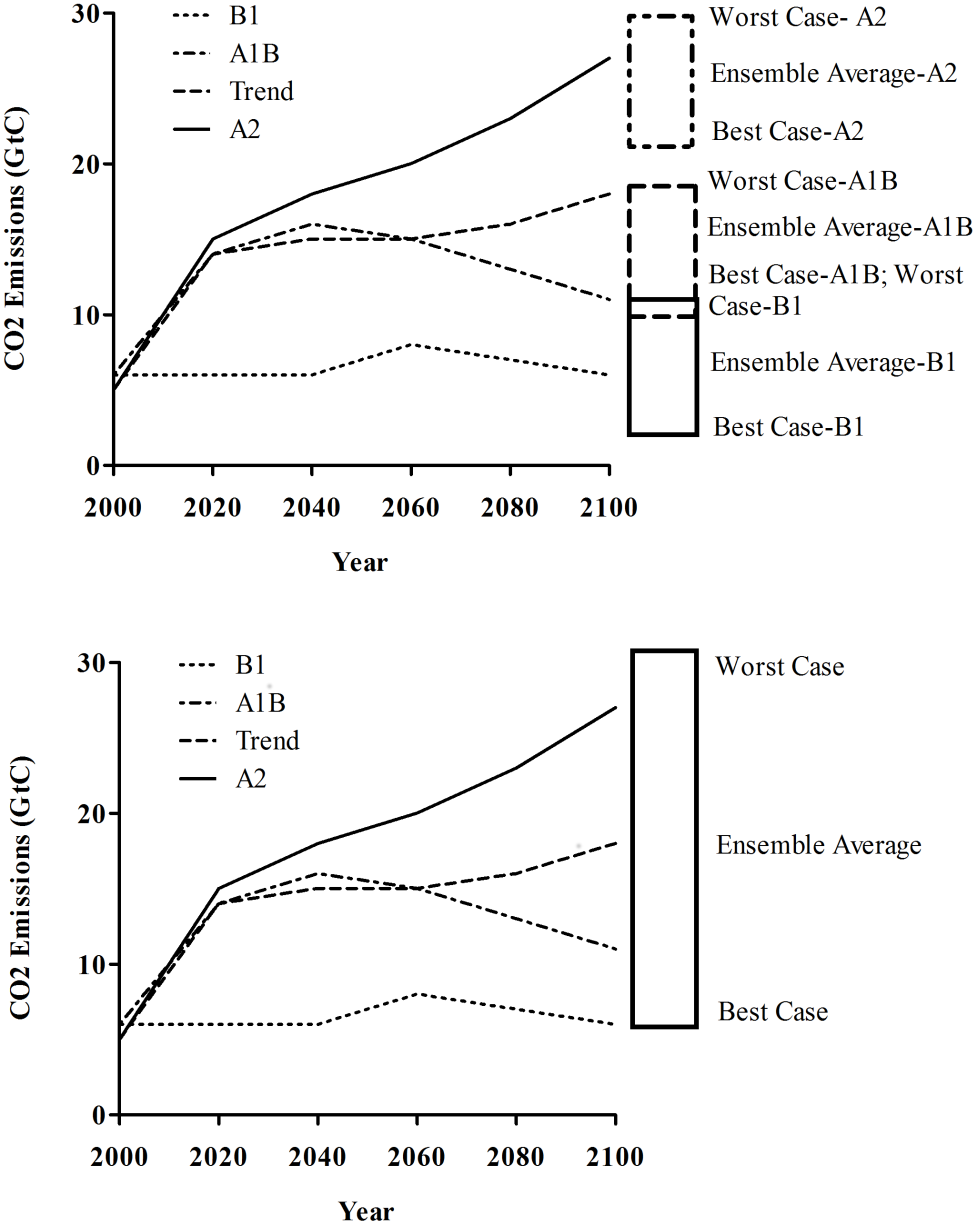
Default and manipulated data arrangement for CO_2_ emission models. Top) Default data arrangement of future values presented by climatewizard.org. For each carbon dioxide emission model (A2, A1B, B1) climate wizard allows the user to obtain values that correspond to the best, worst, and ensemble averages from 16 general circulation models. Bottom) Data arrangement used to obtain future values for our climate change impact assessment. For this assessment, we averaged the best, worst, and average values from 16 general circulation models across all three carbon dioxide emission models.

For our analysis, we obtained six estimates of future predictions: worst case scenario 40 years into the future, best case scenario 40 years into the future, ensemble average 40 years into the future, worst case scenario 70 years into the future, best case scenario 70 years into the future, and ensemble average 70 years into the future. We determined the mean value and variance for each variable by averaging the predictive value over 16 general circulation models and 3 carbon dioxide (CO_2_) emission models. Therefore, each predictive value was based on a vector of 48 future predictions. We chose to average across three CO_2_ emission models for two reasons. First, an independent analysis of changes in variables for each scenario would result in 9 total values (best, worst, and ensemble averages for A2, A1B, and B1) with no clear indication of what best, worst, and ensemble averages would be given some of these values overlap (Figure 1). Within this rationale, evidence suggests humans have released more gigatons of CO_2_ into the atmosphere than the B1 emission scenario predicts, thus the worst case values for the B1 scenario are likely the best case scenario in terms of CO_2_ emissions. We contend it was warranted to have a clear delineation of what was best and worst case scenarios. Averaging the predicted values across all three CO_2_ emission models within the context of best case, worst case, and ensemble averages met this criteria, and subsequently reduced the uncertainty of the estimates because the sample size increased from 16 values (1 from each AOGCM for each CO_2_ model) to 48 values (1 from each AOGCM within 3 CO_2_ models; Figure 1).

If the climatic forecast changed a variable, we applied the change to the corresponding variable in the 11-year data set. For example, if the 40-year best-case forecast called for a 3.03°C increase in winter temperatures (October-March), we applied that increase to the mean winter temperature for our 11-year data set:
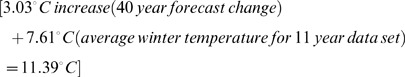



We changed each seasonal variable based on the averaged model predictions across the 6 future scenarios. We then used PROC UNIVARIATE in SAS 9.2 to assess the probability distribution of each predictive variable and then used program R 2.13.1 (The R Foundation for Statistical Computing) to create a vector of 1,000 randomly selected numbers for each seasonal variable based on the mean change of each variable in each scenario, its variance, and the probability distribution. If the data were not uniformly distributed, we log-transformed the data and used a normal probability distribution. We used PROC GAM in SAS 9.2 (Figures S1, S2, S3, S4, S5, S6, S7; Output S1) to assess the relationship between predictor and response variables and then inserted the 1,000 randomly selected variables into the equation that best estimated clutch size, incubation initiation, or nest survival to assess future climatic scenarios.

We extrapolated the predicted values for clutch size, incubation start date, and nest survival (dependent variables) from the equation produced from the interpolated relationship between the dependent and independent variables. Extrapolation of these data are warranted because the goal of our study was to assess the potential changes in reproductive parameters in relation to future climate change using ecological field data collected over ten years [26].

## Results

### Clutch Size

We assessed the relationship between seasonal weather patterns and clutch size for 156 nests. Based on model selection criteria, there was a high degree of model uncertainly among our 9 *a priori* candidate models. The individual parameters winter precipitation (AIC*w* = 0.74) and spring precipitation (AIC*w* = 0.60; Table 1) had the most support. There was moderate support for the global model (AIC*w* = 0.44), but this was likely because this model incorporated the top two competing parameters. Due to the high degree of uncertainty among our models, we obtained the beta coefficients for all variables by model averaging across the 9 *a priori* models. Based on this assessment, the relationship between clutch size and seasonal patterns was:
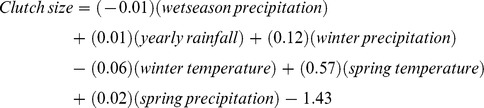



**Table 1:** Output from 9 *a priori* models used to assess the relationship between clutch size and seasonal weather patterns for 156 lesser prairie-chicken nests in Roosevelt County, NM, and Cochran, Hockley, Terry, and Yoakum counties, TX, USA, 2001–2011.

Model	AIC*c*	DeltaAIC*_c_*	AIC*_c_*Weight	Likelihood	*K*	Deviance
Global	1092.94	0	0.44	1	7	1003.72
WinPrecip	1094.53	1.58	0.20	0.92	2	1085.84
SprPrecip	1094.92	1.98	0.16	0.90	2	1088.57
Winter	1095.71	2.76	0.11	0.87	3	1079.57
Spring	1096.86	3.92	0.00	0.82	3	1087.59
WinTemp	1099.65	6.70	0.00	0.71	2	1122.06
YearlyPrecip	1104.41	11.47	0.00	0.56	2	1156.85
WetSeason	1106.60	13.70	0.00	0.50	2	1173.55
SprTemp	1106.69	13.74	0.00	0.50	2	1173.55

WinTemp - Winter Temperature.

WinPrecip - Winter Precipitation.

YearlyPrecip - Yearly Precipitation.

SprTemp - Spring Temperatures.

SprPrecip - Spring Precipitation.

WetSeason - Wet Season Precipitation.

From this equation, the weather model based estimate of mean clutch size is 7.25 eggs per nest.

**Figure 2:**
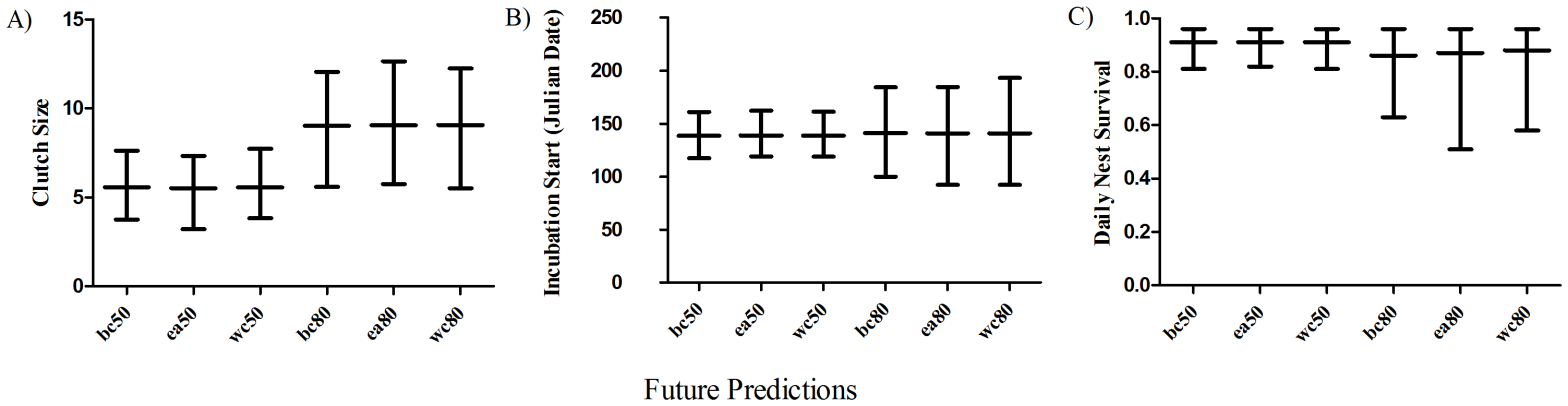
Future predicted values for clutch size, incubation start date, and nest survival. Mean and lower and upper 95% confidence limits of future lesser prairie-chicken A) clutch size, B) incubation start date, and C) nest survival from 1,000 simulations. Each graph presents results from six scenarios: wc50 - worst case scenario, 2050; bc50 - best case scenario, 2050; ea50 - ensemble average scenario, 2050; wc80 - worst case scenario, 2080; bc80 - best case scenario, 2080; ea80 - ensemble average scenario, 2080.

We obtained future predicted values of mean clutch size using model averaged beta parameters across all 9 *a priori* candidate models. The change in value, variance, and predicted value for each seasonal variable under the 40-year and 70-year climate scenarios (Tables 2,3) result in a predicted mean clutch size of 5 eggs per nest by 2050 and 9 eggs by 2080 (Figure 2).

**Table 2:** Mean predicted change for temperature (°C) and precipitation (cm) from 3 climatic forecasts used to predict mean clutch size, incubation initiation, and daily survival rates for lesser prairie-chicken populations in 2050.

Parameter	Scenario	Predicted Change	Variance
Winter Temperatures	Best Case 40	1.98↑	0.63
	Ensemble Average 40	1.98↑	0.64
	Worst Case 40	2.02↑	0.70
Winter Precipitation	Best Case 40	0.01↑	4.08
	Ensemble Average 40	0.01↑	4.08
	Worst Case 40	0.01↑	4.00
Spring Precipitation	Best Case 40	0.12↓	3.20
	Ensemble Average 40	0.13↓	3.23
	Worst Case 40	0.13↓	3.23
Spring Temperature	Best Case 40	2.56↑	0.46
	Ensemble Average 40	2.55↑	0.47
	Worst Case 40	2.57↑	0.46
Yearly Precipitation	Best Case 40	0.04↓	1.94
	Ensemble Average 40	0.04↓	1.57
	Worst Case 40	0.04↓	1.78
Wet Season Precipitation	Best Case 40	0.01↓	2.93
	Ensemble Average 40	0.02↓	2.22
	Worst Case 40	0.03↓	1.71

↑ - Projected Increase.

↓ - Projected Decrease.

**Table 3:** Mean predicted change for temperature (°C) and precipitation (cm) from 3 climatic forecasts used to predict mean clutch size, incubation initiation, and daily nest survival for lesser prairie-chicken populations in 2080.

Parameter	Scenario	Predicted Change	Variance
Winter Temperatures	Best Case 70	2.88↑	1.09
	Ensemble Average 70	3.03↑	1.32
	Worst Case 70	3.03↑	1.33
Winter Precipitation	Best Case 70	0.02 ↓	5.5
	Ensemble Average 70	0.02 ↓	5.5
	Worst Case 70	0.03↓	5.5
Spring Precipitation	Best Case 70	0.17↓	6.00
	Ensemble Average 70	0.17↓	6.29
	Worst Case 70	0.22↓	5.00
Spring Temperature	Best Case 70	3.74↑	1.47
	Ensemble Average 70	3.76↑	1.34
	Worst Case 70	3.78↑	1.35
Yearly Precipitation	Best Case 70	0.05↓	2.29
	Ensemble Average 70	0.05↓	2.08
	Worst Case 70	0.06↓	2.57
Wet Season Precipitation	Best Case 70	0.05↑	5.21
	Ensemble Average 70	0.03↑	5.16
	Worst Case 70	0.05↓	4.03

↑ - Projected Increase.

↓ - Projected Decrease.

### Incubation Start Date

We assessed the relationship between seasonal weather patterns and incubation start date for 207 nests. Based on model selection criteria, there was overwhelming support for the global model (AIC*w*>0.90; Table 4), which incorporates all the parameters; therefore, we did not model average results and we used the beta coefficients from the top competing model.
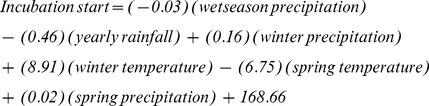



**Table 4:** Output from 9 *a priori* models used to assess the relationship between mean incubation initiation (Julian Date) and seasonal weather patterns for 207 nests in Roosevelt County, NM, and Cochran, Hockley, Terry, and Yoakum counties, TX, USA, 2001–2011.

Model	AIC*c*	DeltaAIC*c*	AIC*c*Weight	Likelihood	*K*	Deviance
Global	2057.82	0	0.98	1	7	19357
Winter	2071.50	13.68	0.00	0.50	3	21541
WinPrecip	2074.20	16.38	0.00	0.44	2	22042
WinTemp	2080.64	22.82	0.00	0.31	2	22739
YearlyPrecip	2085.40	27.66	0.00	0.25	2	23277
WetSeason	2090.65	32.83	0.00	0.19	2	23865
Spring	2098.38	40.56	0.00	0.13	3	24528
SprPrecip	2099.99	42.16	0.00	0.12	2	24966
SprTemp	2103.50	45.68	0.00	0.10	2	25394

WinTemp - Winter Temperature.

WinPrecip - Winter Precipitation.

YearlyPrecip - Yearly Precipitation.

SprTemp - Spring Temperatures.

SprPrecip - Spring Precipitation.

WetSeason - Wet Season Precipitation.

Based on the above equation, the weather model based estimate of incubation initiation was 140 (20-May).

Based on model selection criteria, there was a high degree of model certainty among our 9 *a priori* candidate models. Therefore, we obtained future predicted value of mean incubation initiation using beta coefficients from the top competing model. Based on change in value, variance, and predicted value for each seasonal variable under the 40-year and 70-year scenarios (Tables 2, 3), average incubation initiation is not predicted to change in either 2050 or 2080 (Figure 2).

### Nest Survival

2080 (Figure 2).

### Nest Survival

We assessed nest survival for 229 lesser prairie-chicken nests. Based on model selection criteria (Table 5), there was a high degree of uncertainty for seasonal trends for this analysis. The top three competing models all had ΔAIC*c* values <2. However, based on individual parameter weights, there was strong support for winter temperatures (AIC*w* = 0.70) compared to the other individual variables (Table 5). We obtained predictive beta coefficient estimates by model averaging the beta coefficient using a back-transformed logit-link function and likelihood values:
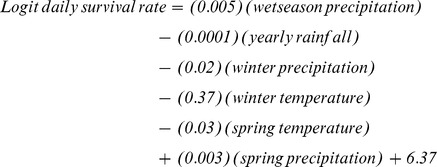



**Table 5:** Output from 9 *a priori* models used to estimate daily nest survival rates for 229 lesser prairie-chicken nests in Roosevelt County, NM, and Cochran, Hockley, Terry, and Yoakum counties, TX, USA, 2001–2011.

Model	AICc	DeltaAICc	AICcWeights	ModelLikelihood	*K*	Deviance
WinTemp	1261.72	0.00	0.28	1.00	2	1257.72
Winter	1261.97	0.24	0.24	0.88	3	1255.96
Global	1262.51	0.78	0.18	0.67	7	1248.48
SprPrecip	1264.78	3.06	0.06	0.21	2	1260.78
Spring	1264.78	3.06	0.06	0.21	2	1260.78
WinPrecip	1265.03	3.31	0.05	0.19	2	1261.03
SprTemp	1265.04	3.32	0.05	0.19	2	1261.04
Wet Season	1265.05	3.32	0.05	0.18	2	1261.04
YearlyPrecip	1431.95	170.22	0.00	0.00	1	1429.95

WinTemp - Winter Temperature.

WinPrecip - Winter Precipitation.

YearlyPrecip - Yearly Precipitation.

SprTemp - Spring Temperatures.

SprPrecip - Spring Precipitation.

WetSeason - Wet Season Precipitation.

Based on the model averaged variables above, the probability of daily nest survival on the study area was 0.98. Assuming a 28 day exposure period (i.e., the incubation period) this translates to a probability of a nest surviving the entire incubation period as 0.57 (0.98^28^).

We obtained future predicted values of daily survival rates using model averaged beta coefficient across all 9 *a priori* candidate models (Tables 2, 3). Average daily survival is predicted to decrease across all six climatic scenarios by 0.50–0.56 from current estimates (Figure 2). Thus, predicted survival of nests through the incubation period to hatching is ≤10% under all 40-year and 70-year scenarios.

## Discussion

Our results suggests with high certainty that nest survival will decrease to a level that is considered below the threshold for population persistence by 2050. The level of precision and repeatability observed in our modeling effort is not common in biological systems. The low standard errors and confidence intervals are ultimately a function of our modeling effort that was weighted to changes in temperature 40 and 70 years into the future that also had high precision. We suggest the results we witnessed for nest survival are reliable because current ambient environmental conditions on the study area often exceed ranges that are considered suitable for stable nest environments (34–40°C; <10% relative humidity; [39]), and temperatures are predicted to increase with subsequent climate change. We do caution that this concern is specifically for lesser prairie chicken populations on the Southern High Plains. Temperatures and relative humidities during nest incubation on the Southern High Plains are, on average, 7°C greater and 7% less, respectively, compared to lesser prairie-chickens in the northeast population (30 year means for Dodge City, Kansas and Lubbock, Texas). The effects of climate change may affect lesser prairie-chickens differently in the northern extent of their range, but we hypothesize that warmer, drier winters and springs, which ultimately influence vegetation and hen condition, will be beyond the threshold for optimal nesting environments on the Southern High Plains.

We found no overwhelming support for any one *a priori* model for clutch size as a function of seasonal weather patterns. Aside from spring temperatures, effect size for each seasonal weather condition in relation to clutch size was relatively weak. These results suggest that weather parameters in general are a weak predictor of clutch size, and other factors may be influencing clutch size to a greater extent. We propose two reasons that may best explain clutch size of lesser prairie-chickens.

First, clutch size is a proximate result of hen condition, which is influenced by environmental conditions [4]. Therefore, we would expect to see larger clutches in years that promote better hen condition. Our results suggest that no such relationship exists, and we hypothesize that visitations to water sources before, during, and after incubation are supplementing egg development and formation during period of drought. Prior knowledge suggested lesser prairie-chickens obtain necessary moisture through food and metabolism [27]. The potential need for free surface water has been generally disregarded, despite information that surface water is used [2], [28]. Based on our ongoing studies on the Southern High Plains, we suspect the contention that free water is not needed by lesser prairie-chicken may be erroneous, at least in terms of reproductive output. Our collaborative studies have photo-documented 1245 different visits to water sources by individual and groups of prairie-chickens during our year-round monitoring of water sources. Males are seen visiting water sources in high numbers from December – June (27–80 detections per month), with few visits during July – November. An estimated 87% of female visits are during the latter part of April, then May – June. This suggests hen use of water sources is strongly associated with egg-formation, laying period, and incubation. It also suggests that water use is not necessarily tied to ambient temperature unless in times of extreme drought; hens rarely visited water sources during the hottest months of July – September, except during 2011 (an extreme drought year), when we recorded 67 different visits. We suggest the proximate reason why we failed to detect a correlation between current and climatic conditions and clutch size is the use of free standing water as an aid to metabolic processes during oogenesis [29], [30].

Hens in the northern extent of lesser prairie-chicken range typically have larger clutches compared to females on the Southern High Plains. Renest attempts are more common in the north [4], [18] compared to our studies. Differences in clutch size, incubation dates, and renest attempts could be attributed to different life history strategies of lesser prairie-chickens between the two ecoregions [4]. On the Southern High Plains, daily nest survival is consistent, clutch sizes are smaller, and renests are rarer compared to populations occupying the northern portion of their occupied range. These patterns suggest that lesser prairie-chickens on the Southern High Plains invest heavily into one nesting attempt, and that may be the ultimate reason we failed to find evidence that clutch size was a function of seasonal weather conditions.

Our results suggest that any one seasonal weather condition is a poor predictor of incubation start date. We did witness strong support for the global model, which incorporated all of the seasonal weather conditions. This suggests that incubation start date was influenced by a combination of seasonal weather conditions, with winter and spring temperatures having the strongest influence on incubation start date. Likewise we found evidence suggesting that winter temperature was the main, strong influence of nest survival among the weather variables considered.

Timing of incubation and nest survival appear to be correlated; previous studies suggest that nests have a higher probability of survival if they are initiated earlier in the year [18], [31]. We hypothesize hot, dry conditions in the winter and spring affect the growth and composition of nest vegetation on our study area, which subsequently influence incubation and nest survival. The two main components of nesting habitat on the Southern High Plains are shrubs and grasses, primarily sand shinnery oak and bluestems (*Andropogon* spp; [32]). Grasses lack the water storage capacity of shrubs and are more reliant on short term precipitation for growth, and when available, hens select bluestems over shrubs for nest sites [32]. Sand shinnery oak roots can extend 10 meters into the soil and enable the shrub to obtain and store water in dry conditions [19]. These and other shrubs are the first plant species on the study area to leaf out [19] because they are less reliant on short-term precipitation. In dry years, we have evidence to suggest that hens select shrubs over grasses. This may explain why we did not find a strong effect of precipitation on reproductive parameters.

We suspect that our findings for incubation start date and nest survival are the result of the El Niño- Southern Oscillation, as the La Niña stage of this oscillation brings warmer, drier winters and springs to the southwest United States [33]. During these years, the probability of drought increases on the Southern High Plains [34], [35], [36], [37]. Lesser prairie-chickens appear to be particularly sensitive to long-term drought [10]. There is additional evidence that lesser prairie-chicken numbers were greatly reduced in the southern population during the extended drought of the 1950s, and took several years to recover to pre-drought numbers [2]. It is important to note that lesser prairie-chickens evolved with drought, and their ability to recover during post-drought years has been documented [9]. However, their ability to recover from long-term (3–10 years) drought on a changing, increasingly fragmented landscape is an unknown, warranted concern for this species.

The 2011 nesting season provides insight to how an extreme La Niña event and subsequent drought affects lesser prairie-chicken reproductive ecology. From 15 October 2010 to 31 August 2011, the total precipitation on the study site was only 2.46 cm (0.97 inches), constituting the worst drought and warmest La Niña event on record. The drought of 2011 was so severe that sand shinnery oak and grasses on the study site did not leaf out, eliminating nesting cover and subsequently delaying nest initiation [38]. Cover provides protection from predators and thermal stress, and when thermal cover is lacking, nests are exposed to the elements (Figure S7).

Preliminary data from nest thermal profiles indicate that temperatures on the ground during the daylight hours exceeded 54.4°C (130°F), and humidity was consistently below 10% in 2011, which is well beyond the threshold for egg viability [39] and nest survival. These data are based on roughly 10% of our nest (22 of 225) from 3 years of study (2010–2012), but indicate there are potential temperature and humidity thresholds for incubating hens. One important aspect of predictive modeling that we did not incorporate into our assessment, but that would benefit future species-level impact assessments, is the incorporation of current and predicted future frequencies of extreme weather events. In some cases, averages may not distinguish between seasonal mean conditions that may have record cold and heat events, and these events may effectively cancel each other out, but still affect demographic parameters. However, this study began in 2001 without the inclusion of climate change as a study objective, and as such, we were ultimately limited by our data in regards to modeling extreme events.

For our extrapolation to be meaningful, we must assume that seasonal weather patterns will have a continued influence on reproductive parameters. Previous studies have demonstrated that reproductive parameters may be influenced by hen age (clutch size, nest survival; [18]) nesting attempt (clutch size; [18]), hen condition (clutch size, nest survival; [4]), and geographic location (clutch size, incubation start, nest survival; [4], [5], [12]). We agree that there are multiple influences on lesser prairie-chicken reproductive parameters, and we contend that clutch size and incubation start date may not be influenced to any extent that is cause for concern in the future. In contrast to these findings, however, is that nest survival is predicted to fall well below the threshold for population persistence [40]. There is sufficient evidence to suggest that lesser prairie-chickens usually experience either booms or busts in reproductive success [16]. The “boom bust” reproductive strategy suggests that lesser prairie-chickens maximize reproductive efforts when conditions are optimal. For the Southern High Plains population, cooler, wetter springs maximize both food and cover for lesser prairie-chickens. Evidence suggests precipitation and temperature influence nest survival [31] and we contend these variables will subsequently continue to influence nest survival in the future.

The role of daily weather on productivity [20] and nest survival [31] of prairie grouse is not well understood, and to the best of our knowledge, this is the first study that has examined the role of seasonal weather conditions on reproductive parameters for lesser prairie-chickens. Although we lack corroboration from other studies, we suggest the main driver of nest survival in the southern population is weather. Fragmentation and habitat destruction have exacerbated the importance of nest survival and reproductive output within small, isolated pockets of lesser prairie-chickens, as the range of lesser prairie-chickens is now restricted to two, unconnected geographic populations.

We attribute the loss of useable space as a primary reason why weather conditions have become a concern for lesser prairie-chickens on our study areas. We hypothesize this relationship will not only continue in the future, but become more prevalent by the persistent loss and fragmentation of current habitats through multiple mechanisms. Since lesser prairie-chickens on the Southern High Plains are isolated to a small portion of shinnery oak-grasslands on the most agriculturally impacted region in the Western Hemisphere, surrounding landscapes that are predominantly cotton fields or livestock pastures dominated by mesquite or juniper trees will not support the species. Therefore, the population of concern currently has no real options in terms of movement and distribution shifts, which exacerbates the troublesome nature of our findings.

Previous studies that have examined shifts in incubation in birds suggest incubation start date will occur earlier due to shifting spring phenology [41], [42], [43], [44], [45]. However, the nesting phenology of lesser prairie-chickens on the study site is predicted to remain stable through 2050 and 2080. These results are based on our inability to find evidence to support seasonal weather variables as a good predictor/influence of incubation start date.

We cannot attribute the 2011 drought and extreme temperatures to climate change, but these types of abnormalities/anomalies and extreme events are expected to occur in greater frequency with subsequent climate change [14]. Current carbon dioxide emissions will ultimately create similar weather patterns that are witnessed during La Niña events, albeit at much higher frequencies [15]. Therefore, under current CO_2_ emission rates, the outlook for nest survival in the southern region of their distribution is not positive. What remains unknown within our assessment is the future relationship between all relevant demographic parameters, habitat availability, climate change, adaptation potential, and the positive benefit of habitat management [46]. Without an entire demographic and landscape assessment, we only have a small piece of a complex puzzle, as the interaction between climate change and main effects (e.g., nest survival) may be offset by the relationship between climate change and interactive effects (e.g. changes in landscape patterns coupled with recruitment).

### Future Directions

Future assessments specifically designed to address climate related objectives for the genera *Tympanuchus* and similar species (Family Phasianidae) may benefit from incorporating 2 important components not used herein. First, climatic predictions using 10, 15 or 20 year intervals would be useful in order to assess when populations may reach critical threshold in terms of population viability. Second, impact assessments that include downscaled weather data from observations collected on site and future predicted values obtained from atmospheric and oceanic general circulation models are well suited to assess short term impacts (5–10 year intervals) at various spatial scales. For existing data sets, the methodologies used herein demonstrate the value of modeling demographic parameters in context of seasonal averages and totals when the ecology of the species suggests an existing relationship. For lesser prairie-chickens, we have demonstrated that on the Southern High Plains, nest survival is influenced by seasonal weather parameters, which is a cause of given future forecasts call for increase probability of drought.

## Supporting Information

Figure S1 **Linear relationship between clutch size and winter precipitation.**
(PNG)

Figure S2 **Linear relationship between clutch size and winter temperature.**
(PNG)

Figure S3 **Linear relationship between incubation start date and winter precipitation.**
(PNG)

Figure S4 **Linear relationship between incubation start date and winter temperature.**
(PNG)

Figure S5 **Linear relationship between nest survival and winter precipitation.**
(JPG)

Figure S6 **Linear relationship between nest survival and winter temperature.**
(JPG)

Figure S7 **Interannual differences in nesting cover**. Differences in nesting cover between a cool, wet spring (2010; top) and hot, dry spring (2011; bottom) on the study area in Roosevelt County, NM, and Cochran, Hockley, Terry, and Yoakum counties, TX, USA, 2001–2011.
(TIF)

Table S1 **Values, means, and standard errors for weather data collected on site.** Values, means, and associated standard errors (in parenthesis) for winter temperature (°C),winter precipitation (cm), yearly precipitation, spring temperatures, spring precipitation, and wet season precipitation used to evaluate lesser prairie-chicken nest survival in Roosevelt County, NM, and Cochran, Hockley, Terry, and Yoakum counties, TX, 2001–2011.
(DOCX)

Table S2 **Names and description of weather models developed to assess the effects of seasonal weather patterns on lesser prairie-chicken reproductive parameters.** Description, names, and number of parameters for nine *a priori* seasonal weather models used to assess daily nest survival, clutch size, and incubation initiation in Roosevelt County, NM, and Cochran, Hockley, Terry, and Yoakum counties, TX, USA, 2001–2011.
(DOCX)

Table S3 **Detailed description of three greenhouse gas emission scenarios used to obtain predicted values of climatic variables.**
(DOCX)

Output S1 **Output from Proc GAM in SAS 9.2.**
(MHT)
