# Supplementary figures and images for: The Predicted Influence of Climate Change on Lesser Prairie-Chicken Reproductive Parameters
Source: PLoS One. 2013 Jul 11;8(7):e68225. doi: 10.1371/journal.pone.0068225 (PMC3708951; doi:10.1371/journal.pone.0068225)

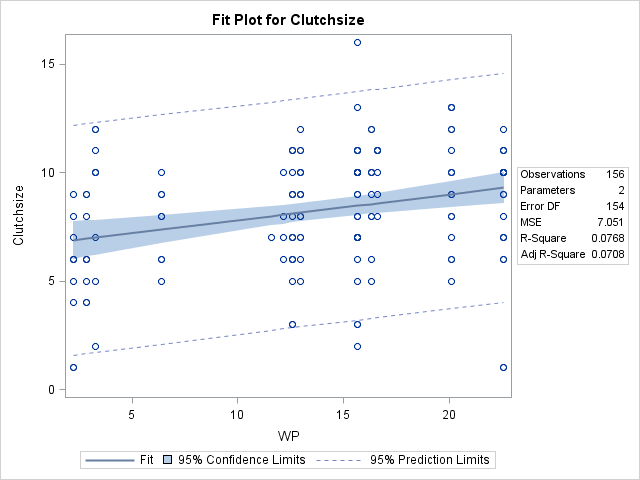

Supplement: Figure S1 — Linear relationship between clutch size and winter precipitation. (PNG) [file pone.0068225.s001.png]

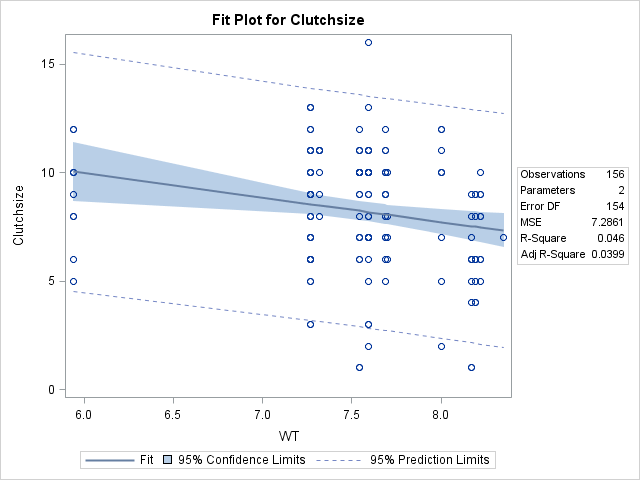

Supplement: Figure S2 — Linear relationship between clutch size and winter temperature. (PNG) [file pone.0068225.s002.png]

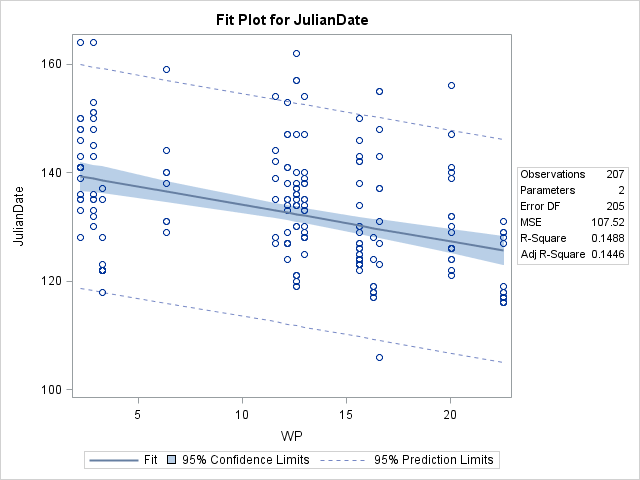

Supplement: Figure S3 — Linear relationship between incubation start date and winter precipitation. (PNG) [file pone.0068225.s003.png]

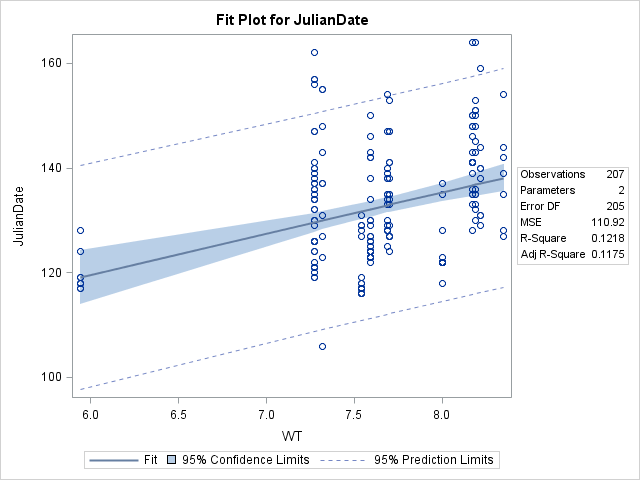

Supplement: Figure S4 — Linear relationship between incubation start date and winter temperature. (PNG) [file pone.0068225.s004.png]

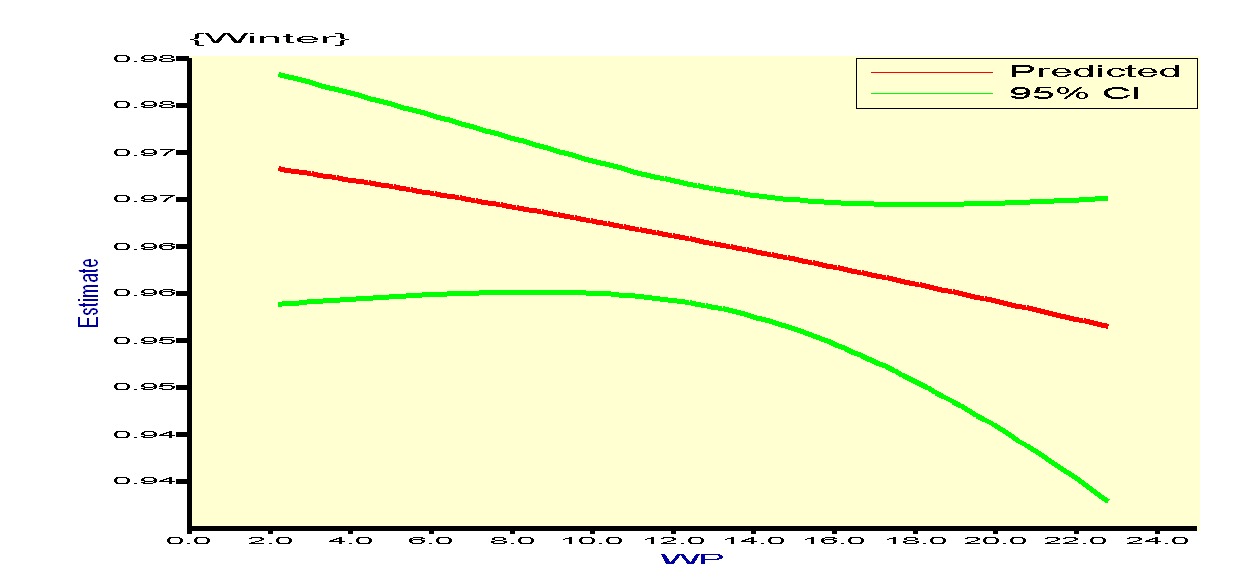

Supplement: Figure S5 — Linear relationship between nest survival and winter precipitation. (JPG) [file pone.0068225.s005.jpg]

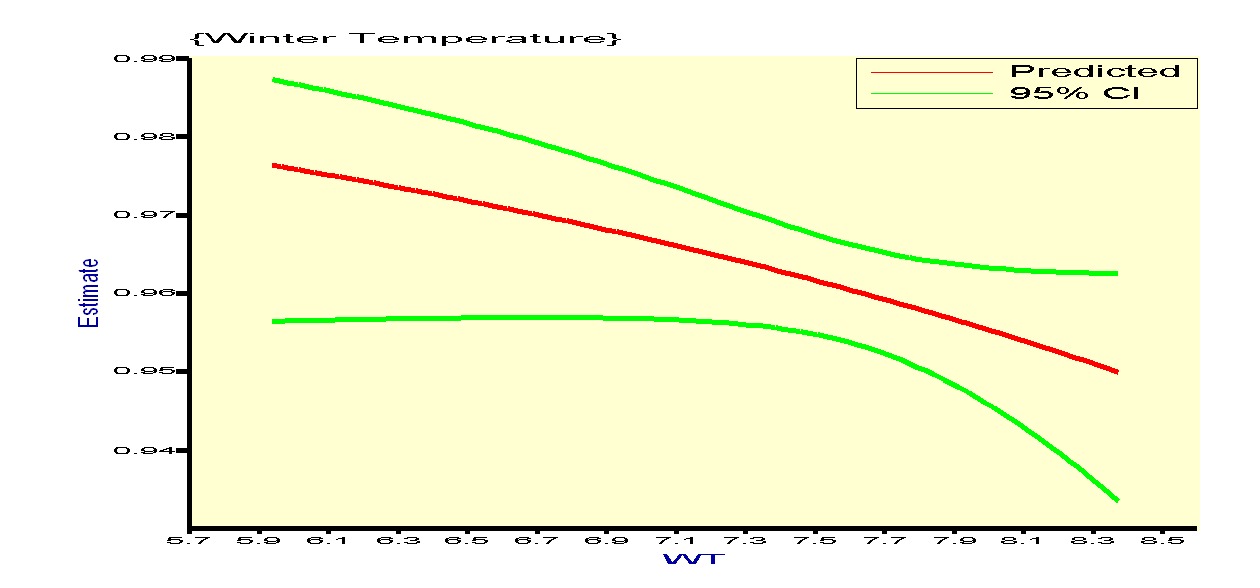

Supplement: Figure S6 — Linear relationship between nest survival and winter temperature. (JPG) [file pone.0068225.s006.jpg]

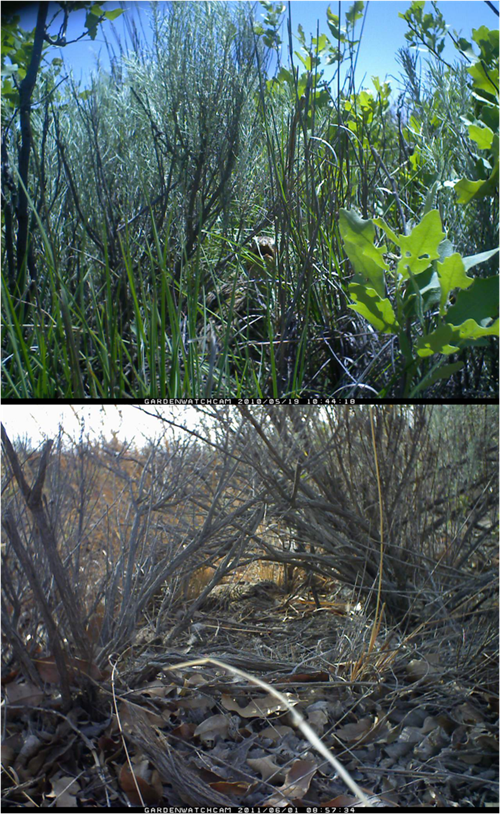

Supplement: Figure S7 — Interannual differences in nesting cover. Differences in nesting cover between a cool, wet spring (2010; top) and hot, dry spring (2011; bottom) on the study area in Roosevelt County, NM, and Cochran, Hockley, Terry, and Yoakum counties, TX, USA, 2001–2011. (TIF) [file pone.0068225.s007.tif]
