# Supplementary material for: The Predicted Influence of Climate Change on Lesser Prairie-Chicken Reproductive Parameters
Source: PLoS One. 2013 Jul 11;8(7):e68225. doi: 10.1371/journal.pone.0068225 (PMC3708951; doi:10.1371/journal.pone.0068225)
Supplement: Table S1 — Values, means, and standard errors for weather data collected on site. Values, means, and associated standard errors (in parenthesis) for winter temperature (°C),winter precipitation (cm), yearly precipitation, spring temperatures, spring precipitation, and wet season precipitation used to evaluate lesser prairie-chicken nest survival in Roosevelt County, NM, and Cochran, Hockley, Terry, and Yoakum counties, TX, 2001–2011. (DOCX) [file pone.0068225.s008.docx]

| Site | Year | WinTemp | WinPrecip | YearlyPrecip | SprTemp | SprPrecip | WetSeason |
| --- | --- | --- | --- | --- | --- | --- | --- |
| NM | 2001 | 7.69 | 12.98 | 47.68 | 12.61 | 1.78 | 28.65 |
| NM | 2002 | 7.59 | 15.65 | 45.01 | 12.59 | 9.32 | 20.07 |
| NM | 2003 | 7.27 | 12.6 | 34.14 | 12.88 | 0.33 | 30.28 |
| NM | 2004 | 8.35 | 11.58 | 57.91 | 12.68 | 16.51 | 30.33 |
| NM | 2005 | 7.54 | 22.58 | 86.79 | 11.06 | 1.93 | 54.81 |
| NM | 2006 | 8.00 | 3.25 | 22.33 | 13.01 | 6.13 | 5.73 |
| NM | 2007 | 7.32 | 16.61 | 48.59 | 12.93 | 14.9 | 12.81 |
| NM | 2008 | 8.17 | 2.21 | 37.29 | 12.22 | 0.65 | 6.28 |
| TX | 2008 | 8.19 | 2.84 | 28.80 | 12.72 | 0.90 | 6.94 |
| NM | 2009 | 7.70 | 12.17 | 43.66 | 13.01 | 5.99 | 10.35 |
| TX | 2009 | 8.22 | 6.38 | 32.87 | 13.66 | 4.04 | 7.95 |
| NM | 2010 | 7.27 | 20.09 | 53.37 | 11.65 | 13.82 | 12.97 |
| TX | 2010 | 5.94 | 16.33 | 46.74 | 12.17 | 17.61 | 5.87 |
| TX | 2011* | 7.46 | 5.33 | 39.09 | 15.30 | 0.36 | 29.56 |
| Mean |  | 7.62(0.16) | 11.47(1.75) | 44.59(4.15) | 12.75(0.25) | 6.73(1.73) | 18.75(3.83) |

* We observed no nest initiation attempts in New Mexico in 2011. See Table 2 for seasonal descriptions.

WinTemp - Winter Temperature

WinPrecip - Winter Precipitation

YearlyPrecip - Yearly Precipitation

SprTemp - Spring Temperatures

SprPrecip - Spring Precipitation

WetSeason - Wet Season Precipitation
