# Supplementary material for: The Predicted Influence of Climate Change on Lesser Prairie-Chicken Reproductive Parameters
Source: PLoS One. 2013 Jul 11;8(7):e68225. doi: 10.1371/journal.pone.0068225 (PMC3708951; doi:10.1371/journal.pone.0068225)
Supplement: Table S2 — Names and description of weather models developed to assess the effects of seasonal weather patterns on lesser prairie-chicken reproductive parameters. Description, names, and number of parameters for nine a priori seasonal weather models used to assess daily nest survival, clutch size, and incubation initiation in Roosevelt County, NM, and Cochran, Hockley, Terry, and Yoakum counties, TX, USA, 2001–2011. (DOCX) [file pone.0068225.s009.docx]

| Model | Name | Description |
| --- | --- | --- |
| S _Global_ | Yearly | Parameter is a function of all seasonal trends combined from previous and corresponding years |
| S _Yearly Precipitation_ | Yearly Precipitation | Parameter is a function of total precipitation from June-April for corresponding year |
| S _Wet Season Precipitation_ | Wet Season Precipitation | Parameter is a function of total precipitation from July-October of previous year |
| S _Spring Temps and Precip_ | Spring | Parameter is a function of average temperature and total precipitation l for March and April of corresponding year |
| S _Spring Temperatures_ | Spring Temperatures | Parameter is a function of average temperature for March and April of corresponding year |
| S _Spring Precipitation_ | Spring Precipitation | Parameter is a function of total precipitation for March and April of corresponding year |
| S _Winter Temps and Precip_ | Winter | Parameter is a function of average temperature and total rainfall for October- December of previous year and January-February of corresponding year |
| S _Winter Temperatures_ | Winter Temperatures | Parameter is a function of average temperature for October- December of previous year and January-February of corresponding year |
| S _Winter Precipitation_ | Winter Precipitation | Parameter is a function of total precipitation for October- December of previous year and January-February of corresponding year |
