# Supplementary material for: The Predicted Influence of Climate Change on Lesser Prairie-Chicken Reproductive Parameters
Source: PLoS One. 2013 Jul 11;8(7):e68225. doi: 10.1371/journal.pone.0068225 (PMC3708951; doi:10.1371/journal.pone.0068225)
Supplement: Table S3 — Detailed description of three greenhouse gas emission scenarios used to obtain predicted values of climatic variables. (DOCX) [file pone.0068225.s010.docx]

| Model | Description |
| --- | --- |
| A1B | Assumes a world of very rapid economic growth, global population that peaks in mid-century, and rapid introduction of new and more efficient technologies with a balance of fossil intensive and non-fossil energy sources |
| A2 | Heterogeneous world with high population growth, slow economic development and slow technological change |
| B1 | Intermediate population and economic growth, emphasizes local solutions to economic, social, and environmental sustainability |
